# Introduction of Laparoscopy in an Urban High-Volume Sub-Saharan Trauma Centre

**DOI:** 10.1007/s00268-023-06980-z

**Published:** 2023-03-30

**Authors:** Shumani Makhadi, Megan Lubout, Maeyane S. Moeng

**Affiliations:** Department of Surgery, School of Clinical Medicine, Faculty of Health Sciences, University of the Witwatersrand, Johannesburg, 2193 South Africa

## Abstract

**Introduction:**

Trauma is a major disease burden in low and middle-income countries like South Africa. Abdominal trauma is one of the leading reasons for emergency surgery. The standard of care for these patients is a laparotomy. In selected trauma patients, laparoscopy has both diagnostic and therapeutic usage. The trauma burden and the number of cases seen in a busy trauma unit make laparoscopy challenging.

**Aim:**

We wanted to describe our journey with laparoscopy in the management of abdominal trauma in a busy urban trauma unit in Johannesburg, South Africa.

**Methods:**

We reviewed all trauma patients who underwent diagnostic laparoscopy (DL) or therapeutic laparoscopy (TL) between 01 January 2017 and 31 October 2020 for blunt and penetrating abdominal trauma. The demographic data, indications for laparoscopy, injuries identified, procedures performed, intraoperative laparoscopic complications, conversion to laparotomy, morbidity, and mortality were evaluated.

**Results:**

A total of 54 patients who had laparoscopy were included in the study. The median age was 29 years (IQR 25–25). Most injuries were penetrating 85.2% (*n* = 46/54) and 14.8% blunt trauma. Most patients were males, 94.4% (*n* = 51/54). Indications for laparoscopy included diaphragm evaluation (40.7%), pneumoperitoneum for evaluation of potential bowel injury (16.7%), free fluid with no evidence of solid organ injury (12.9%) and colostomy (5.5%). There were 8 (14.8%) cases converted to laparotomy. There were no missed injuries or mortality in the study group.

**Conclusion:**

Laparoscopy in selected trauma patients is safe even in a busy trauma unit. It is associated with less morbidity and shortened hospital length of stay.

## Introduction

Trauma is the leading cause of death in adults under the age of 35 [1]. It is a significant challenge and financial burden to healthcare systems [2]. The distribution of blunt and penetrating injuries differs according to geographical location, with developed countries treating more blunt than penetrating injuries [1–4]. At least 9–14% of trauma involves the abdominal cavity [2]. Laparotomy is the standard approach for patients needing surgical intervention with morbidity rates of 20–41%, especially in non-therapeutic laparotomies [3–5].

Advances have been made in laparoscopic technology, and surgeons are embracing the usage of laparoscopy for various diseases [6–8]. Studies have shown that laparoscopy for trauma is feasible [7, 8]. There is a lack of randomised control trials to describe the role of laparoscopy in trauma [9]. Short first described exploratory “coelioscopy” for trauma patients in 1925 [10]. The initial experiences with laparoscopy in abdominal disorders and trauma dates from the 1960s, and laparoscopy then made its way into clinical practice [10]. However, not much progress has been made in trauma laparoscopy compared to gastrointestinal surgery [7, 8].

When used in select trauma patients, trauma laparoscopy has been shown to reduce morbidity and postoperative pain, decrease overall cost and length of hospital stay, and allow for an earlier return to work [7, 11]. However, there are concerns about the risk of missing injuries with laparoscopy [7, 12, 13]. Laparoscopy is contraindicated in haemodynamically unstable patients and patients with traumatic brain injuries because of the effects of pneumoperitoneum on cardiovascular physiology and intracranial pressure [12].

Despite these limitations, a large and growing body of literature addresses trauma laparoscopy [5, 7, 9, 11–13]. A true consensus among trauma surgeons regarding its use is yet to be reached, especially as the role of laparoscopy continues to evolve [7, 12, 13]. Koto et al. in South Africa have shown the safety of laparoscopy in trauma and its benefits [7].

We see significant penetrating abdominal trauma in our institution [14, 15]. The emergency workload further adds pressure on theatre availability time. The traditional Trauma surgeon may not necessarily possess the expertise to perform a safe laparoscopy, thus hindering the time required for the learning curve when introducing laparoscopy in trauma [16].

**Fig. 1:**
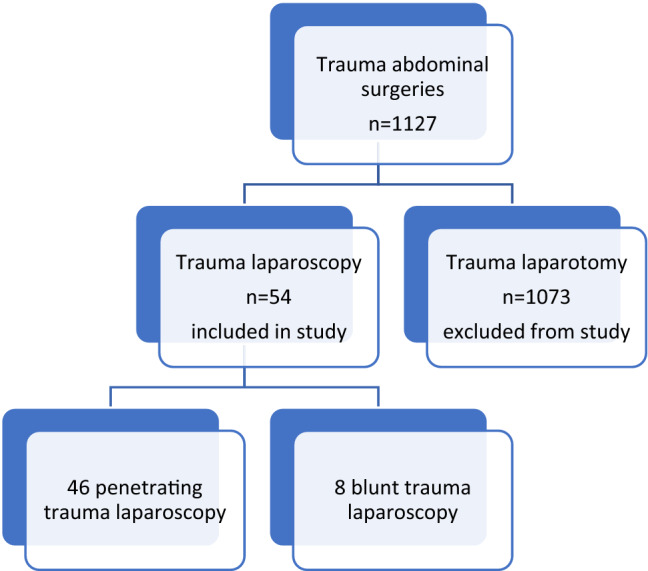
Study diagram

The purpose of the study was to reflect on our journey with the introduction of laparoscopy in our institution. We also wanted to explore the safety of laparoscopy in both blunt and penetrating trauma in a high-volume urban trauma centre (Fig. 1).

## Methods

We performed a retrospective review of all trauma patients who underwent diagnostic laparoscopy (DL) or therapeutic laparoscopy (TL) between January 2017 and October 2020 at Charlotte Maxeke Johannesburg Academic Hospital (CMJAH), a busy urban trauma centre in Johannesburg, South Africa.

Patients were identified from the institution’s trauma database, and only haemodynamically stable patients were included in the study. Data on patient demographics (age, sex), vital signs on presentation (systolic blood pressure, pulse), injury mechanism (penetrating versus blunt), indications for laparoscopy, injuries identified, and outcomes were collected. Primary outcomes included the successful completion rate of the procedure, rate of conversion to laparotomy and rates of missed injury. Secondary outcomes included laparoscopy-associated complications, hospital LOS, pneumonia, superficial site infection (SSI), and in-hospital mortality rates.

We excluded patients with isolated right upper quadrant penetrating injuries as it’s the units protocol to manage them nonoperatively.

Non-therapeutic laparoscopy was considered when no injury was identified that required intervention. “Missed injuries” were defined as intraperitoneal injuries diagnosed after conversion to open laparotomy or diagnosed post-operatively.

Ethics approval was obtained from the Human Research Ethics Committee of the University of the Witwatersrand. The ethics approval number is M201197. Permission to do the study was also obtained from the CMJAH hospital management.

### Statistical analyses

Means (standard deviations, SD) and medians (interquartile ranges, IQR) were calculated for normally distributed and skewed continuous variables, respectively. Frequencies and percentages were used to describe distributions of categorical variables. All analyses were done using STATA version 15. Continuous variables were first tested for normality using the Shapiro–Wilk test. Fisher’s exact test was used to test the significance of the relationship between categorical variables. A *p *value of < 0.05 was considered statistically significant.

## Results

There were 1127 emergency abdominal trauma operations (laparotomies and laparoscopies) performed in the period, of which 54 (4.8%) had laparoscopy and were included in the study. The median age was 29 years (IQR 25–25). Most injuries were penetrating 85.2% (*n* = 46/54) and 14.8% blunt trauma. Among patients with penetrating trauma, 80.4% (*n* = 37/46) sustained stab wounds, and 19.6% (*n* = 9/46) sustained gunshot wounds. The location of injuries in penetrating trauma were anterior (*n* = 32/46), flank (*n* = 8/46) and back (*n* = 6/46). Majority of patients were males, 94.4% (*n* = 51/54).

The most common indications for laparoscopy included diaphragm evaluation (40.7%), pneumoperitoneum for evaluation of potential bowel injury (16.7%), free fluid with no evidence of solid organ injury (12.9%), and colostomy (5.5%) see Table 1. Twelve percent of patients were done in the elective board due to lack of emergency theatre time.

**Table 1:** Indications for exploration

Indication	Number of patients(percentage)
Diaphragm injury	22 (40.7%)
Pneumoperitoneum	9 (16.7%)
Free fluid without a solid organ injury	7 (12.9%)
Colostomy	3 (5.5%)
Bladder injury	2 (3.7%)
Others	11 (20.4%)
Retroperitoneal haematoma	4 (7.4%)
Haemoperitonuem	4 (7.4%)
Bronchopleural fistula	2 (3.7%)
Omental evisceration	1 (1.8%)

The study group had 9 (16.7%) non-therapeutic laparoscopic explorations. Seven had penetrating trauma, and two had blunt injuries.

There were 8 (14.8%) cases converted to laparotomy. Indications for conversion included: the need for better visualisation (*n* = 6), inexperience in dealing with injury (*n* = 1), and bleeding spleen during a diaphragm repair (*n* = 1).

There were 22 diaphragm repairs, with two converted to open repairs. The stomach, small bowel, and colon were injured 2, 3 and 4 times, respectively. Of which 2 stomach injuries (3.7%), 2 small bowel injuries (3.7%) and 2 colon injuries (3.7%) were converted to laparotomy. There were two laparoscopic bladder repairs after blunt trauma (see Table 2).

**Table 2:** Organs injured with conversion rate to laparotomy

Organ (converted to laparotomy/total number)		Percentage
Diaphragm	2/22	9.1
Spleen	1/8	12.5
Liver	1/5	20.0
Colon	2/4	50.0
Small bowel	2/3	66.6
Stomach	2/2	100.0
Bladder	0/2	0.0

There were no missed injuries in all the patients. There was one morbidity of a right-sided haemothorax, diaphragm and liver injury patient who required a relook laparoscopy to re-fix the diaphragm and thoracotomy for a pulmonary toilet. One patient had a relook laparoscopy for a liver injury that needed repositioning of a pencil drain for a bile leak.

Hospital LOS was shorter in the laparoscopy group vs laparoscopy converted to laparotomy group (3 vs 7 days). No in-hospital mortalities occurred in this cohort.

## Discussion

Laparoscopy has advanced in General surgery, but not much progress has been made in trauma surgery [7, 8, 16]. Laparoscopy has evolved to include natural orifice surgery and robotic surgery [17]. Despite advances in laparoscopy, it is still a challenge to accurately identify patients with hollow viscus injuries after blunt and penetrating trauma [17–19]. Most of the patients in our cohort were males, which is in keeping with previous studies done in trauma [4, 19, 20]. The laparoscopy cohort at our institution represents 4.8% of emergency abdominal operations during the study period; this is a small sample compared to patients who had laparotomies. Theatre access challenges, delays and patients’ physiology precluded most patients from being offered laparoscopic surgery [20].

The protocol of the unit is to do an intravenous contrast-enhanced CT abdomen patient with trauma to the abdomen in the absence of clear indications for laparotomy, namely: peritonitis, free air on CXR, blood on the nasogastric tube, blood per rectum, omental/bowel evisceration, transabdominal gunshot wounds crossing the midline [21, 22]. The CT scan assists in determining the need for exploring the retroperitoneum, which can be challenging laparoscopically. CT scan is sometimes unreliable in identifying hollow viscus injuries, in contrast to a diagnostic laparoscopy with a reported sensitivity, specificity and accuracy approaching 100% [23–25]. Laparoscopy also offers the advantage of being therapeutic. Approximately half of the patients in the study had CT scans done. This CT scan approach may have reduced the number of patients for who other institutions could have offered laparoscopy.

There were no missed injuries in our study cohort. Several studies from the 1990s showed that patients undergoing diagnostic laparoscopy (DL) had a high rate of missed injury (41%) [7, 11]. However, as laparoscopic instruments and expertise improve, the reported rates of missed injuries have decreased to as low as 0.12% [7, 11, 15]. This decline has been ascribed to the introduction of a systematic examination in trauma laparoscopy [7]. The low rate of missed injuries in our study can be explained by using CT scans and laparoscopy in combination, especially in patients with high-energy mechanisms. The exploration for injuries followed the standard examination described in other studies [7, 11]. Most of our patients have stab wounds which are low energy and can also attribute to the low number of missed injuries.

Laparoscopy in trauma has both diagnostic and therapeutic use and has demonstrated that when used in selected trauma patients [7, 11, 25]. It reduces morbidity, mortality, postoperative pain, surgical wound infection rate, hospital stay, and hospital costs [4, 7, 16]. Laparoscopic explorations in trauma can also be used as part of Enhanced Recovery after Surgery (ERAS) strategies. It also reduces the number of non-therapeutic laparotomies [25]. The hospital LOS was shorter in the laparoscopy group compared to the patients converted to laparotomy in our study (3 vs 7 days). The morbidity was also low in the cohort. The short hospital LOS and low morbidity in patients undergoing laparoscopy allowed the early discharge of patients post-surgery in our patients.

Our conversion rate is comparable to other studies [7, 26, 27]. Most of our patients were stab wounds which can explain the low conversion rate. Conversions to laparoscopy in our cohort were common in the setting of bowel injuries, especially the stomach, due to the fear of missing other injuries. Those comfortable with laparoscopy often continued with laparoscopy to exclude and fix injuries identified laparoscopically. A laparoscopic-assisted approach was also an option in certain circumstances to improve and boost confidence in laparoscopy in those not comfortable [27].

Our study demonstrates the advantages of laparoscopy in evaluating bowel injuries. We had no missed injuries in this cohort. It reduced the number of non-therapeutic laparotomies during the period of the study.

### Limitations of the study

The retrospective design and single-centre data have a potential for bias. The sample size is small, with only 4.8% of cases receiving laparoscopy. This may reflect the selection bias of more severe patients being treated at the centre and possible logistic factors delaying the full integration of laparoscopy in the unit. Reliance on CT scans in doubtful cases may have further reduced the number of patients requiring diagnostic or even therapeutic laparoscopy.

## Conclusion

Laparoscopy in selected blunt and penetrating trauma patients is safe even in a busy trauma unit. It is associated with less morbidity and shortened hospital length of stay. Larger trials are required to increase further a rational use of laparoscopy beyond diagnostic capabilities in a high-volume Trauma unit.
